# Anti-staphylococcal activity of the auranofin-analogous PEt_3_AuCl: antibacterial, anti-biofilm and anti-virulence effect on clinically relevant staphylococci

**DOI:** 10.3389/fcimb.2026.1794590

**Published:** 2026-04-10

**Authors:** Diletta Mazzantini, Beatrice Amato, Stefano Zineddu, José Aleixo de Azevedo-França, Giuseppantonio Maisetta, Emilia Ghelardi, Semih Esin, Luigi Messori, Giovanna Batoni

**Affiliations:** 1 Department of Translational Research and New Technologies in Medicine and Surgery, University of Pisa, Pisa, Italy; 2 Department of Chemistry “Ugo Schiff”, University of Florence, Florence, Italy

**Keywords:** AF-Cl, biofilm, gold-compounds, staphylococci, virulence

## Abstract

**Objectives:**

Gold(I) complexes, such as the drug Auranofin (AF) which is approved for treating rheumatoid arthritis, have attracted significant interest as a potential treatment for bacterial infections due to their promising, broad-spectrum antimicrobial activity. In this study, we investigated the anti-staphylococcal activity of three AF analogues [PEt_3_AuCl (AF-Cl), PEt_3_AuI (AF-I) and PPh_3_AuCl (TPP-AuCl)] with the aim of discovering new weapons in the fight against antibiotic resistance.

**Methods:**

The antimicrobial activity and cytotoxicity of the gold compounds were evaluated by broth microdilution and the WST-1 assay, respectively. Time-kill assays were used to investigate killing kinetics, and the crystal violet (CV) assay was used to evaluate biofilm formation. Eradication of mature biofilms was assessed using the crystal violet assay, a plate count of biofilm-associated cells and scanning electron microscopy. The anti-virulence effect was tested by the hemolysis and agar diffusion assays.

**Results:**

All of the AF analogues were active against staphylococci, including antibiotic-resistant strains, with minimum inhibitory concentrations (MICs) ranging from 0.063 to 4 µg/mL. Additionally, they exhibited lower toxicity towards the A549 lung cell line and the spontaneously immortalized human keratinocyte line HaCaT than AF. AF-Cl was identified as the most promising compound and was selected for further biological investigations. Time-kill experiments revealed that AF-Cl was rapidly bactericidal against clinical staphylococci, causing at least a 3-log reduction in the number of viable cells within six hours. At sub-inhibitory concentrations, the compound inhibited biofilm formation and reduced the secretion of hemolysins and phospholipases, representing key virulence factors in *S. aureus* infections. Furthermore, AF-Cl was able to eradicate mature *S. aureus* biofilms at non-cytotoxic concentrations.

**Conclusion:**

Overall, our findings highlight the potential of AF-Cl as a promising candidate for treating staphylococcal infections, including those caused by antibiotic-resistant strains. In addition, the compound exhibited anti-biofilm and anti-virulence properties, which could be advantageous in treating toxin-mediated and biofilm-associated staphylococcal diseases.

## 1 Introduction

In the current century, antimicrobial resistance (AMR) is one of the most critical public health concerns. It is estimated that antimicrobial-resistant infections cause more than 29,000 and 35,000 deaths per year in the USA and Europe, respectively (Cassini et al., 2019; European Center for Disease Prevention and Control, 2023; Weist and Hogberg, 2016). Furthermore, an increase in the AMR burden was observed following the SARS-CoV-2 pandemic (European Center for Disease Prevention and Control, 2023). In 2024, the World Health Organization updated the list of AR pathogens for which new antibiotics are urgently needed. Among them is methicillin-resistant *Staphylococcus aureus*, which has been given a ‘high priority status’ due to its high morbidity and mortality rates, and the associated healthcare costs (World Health Organization, 2024). *S. aureus* often causes hospital- and community-acquired infections, including wound, bone, eye, soft tissue, urinary, respiratory, and systemic infections. This is due to its ability to produce a variety of virulence factors (e.g. adhesion proteins, toxins and exoenzymes), to evade the host immune response, and to form robust biofilms on human tissues and medical devices (Cheung et al., 2021; Lathram and Radka, 2025; Touaitia et al., 2025). Biofilms are highly resistant to chemicals, antimicrobials, and immune cells, thereby enhancing the survival of *S. aureus* and contributing to recalcitrant, biofilm-related infections that are extremely difficult to treat (Beenken and Smeltzer, 2025). In addition to *S. aureus*, *S. epidermidis* is frequently the causative agent of opportunistic infections in humans and has been shown to possess moderate virulence potential and a high level of AMR (Burke et al., 2024).

The rise of AMR has made the development of new therapeutic options for treating AMR infections mandatory. Over the last decade, natural molecules, nanoparticles, bacteriophages, monoclonal antibodies, antimicrobial peptides, probiotics and silver- and gold-based compounds have been considered as alternatives to conventional antibiotics (Berger and Loewy, 2024; Ratia et al., 2022). Among these, Auranofin (AF) has attracted considerable attention. AF is an anti-inflammatory drug that was approved by the US Food and Drug Administration (FDA) for the treatment of rheumatoid arthritis in 1985 (Coscione et al., 2024; Yamashita, 2021). Its structure consists of a gold(I) center that is connected to a triethylphosphine group and a thiosugar tetraacetate moiety (Coscione et al., 2024). Several reports have highlighted AF’s significant antimicrobial activity, particularly against Gram-positive microbes, suggesting its potential as an effective antimicrobial agent (Coscione et al., 2024; Ma et al., 2025). In bacteria, the drug primarily acts by inhibiting thioredoxin reductase, an enzyme that plays a role in maintaining redox homeostasis, scavenging reactive oxygen species (ROS), and detoxifying drugs, radicals, and oxidants (Coscione et al., 2024; Quadros Barsé et al., 2024). Notably, AF has also been shown to modulate protein, DNA and cell wall synthesis, as well as the production of certain virulence factors. This suggests that AF acts via multiple targets (Quadros Barsé et al., 2024; Thangamani et al., 2016). Over time, several AF analogs have been synthesized by replacing its thiosugar tetraacetate or triethylphosphine ligands with other functional groups, with the aim to improve its antimicrobial or pharmacological properties (Epstein et al., 2019; Ferretti et al., 2025; Marzo et al., 2018; Maydaniuk et al., 2021).

This study investigated the anti-staphylococcal activity and cytotoxicity on human cells of three gold(I) complexes inspired by auranofin (AF), namely PEt_3_AuCl (AF-Cl), PEt_3_AuI (AF-I), and PPh_3_AuCl (TPP-AuCl). These gold(I) complexes structurally differ from AF due to variations in the nature of the two gold ligands. In AF-Cl and AF-I, the thiosugar ligand of AF is replaced by a chloride or iodide ligand, respectively, while the phosphine ligand remains unchanged. In TPP-AuCl, the gold(I) center is linearly coordinated to triethylphosphine and chloride. To validate the potential use of AF-Cl, the most promising compound in this series, for treating biofilm- and toxin-related *S. aureus* infections, we also evaluated its anti-biofilm and anti-virulence properties.

## 2 Materials and methods

### 2.1 Bacterial strains and culture conditions

*S. aureus* and *S. epidermidis* strains used in this study and their resistance profiles are listed in **Table 1**. *S. aureus* ATCC 6538 and *S. epidermidis* ATCC 35984 were purchased from LGC Standards (Milan, Italy). *S. aureus* W4, *S. epidermidis* CI-1, and *S. epidermidis* CI-2 were isolated from clinical samples in previous studies (Kaya et al., 2024; Mazzantini et al., 2024b). All these *Staphylococcus* strains were previously characterized for their resistance to antibiotics (Kaya et al., 2024; Mazzantini et al., 2024b)*. S. aureus* H1, *S. aureus* H2, and *S. epidermidis* ME120121 were isolated from blood cultures at the Microbiology Unit of Pisa University Hospital and was identified with scores ≥ 2.00 by Matrix Assisted Laser Desorption Ionization-Time of Flight Mass Spectrometry (MALDI-TOF MS) using a MALDI Microflex LT Mass Spectrometer (Bruker Daltonics, Bremen, Germany) as previously described (Ghelardi et al., 2023). The antibiotic susceptibility profile of these strains was determined by broth microdilution using the ITGP100 panels (Bruker Daltonics, Bremen, Germany) as previously reported (Mazzantini et al., 2024b). The Clinical Breakpoints for *Staphylococcus* spp. (version 16.0; The European Committee on Antimicrobial Susceptibility Testing, 2026) were adopted for categorizing *S. aureus* H2 and *S. epidermidis* ME120121 as susceptible (S), increased exposure (I), or resistant (R).

**Table 1:** Bacterial strains used in this study.

Strain	Features	Resistance profile^a^	Reference
*S. aureus* ATCC 6538	Reference strain	Susceptible to all the tested antibiotics	Mazzantini et al., 2024a
*S. aureus* W4	Clinical isolate	Resistant to AMP, CIP, CLI, ERY, FOF, GEN, OXA, PEN, and TET	Kaya et al., 2024
*S. aureus* H1	Clinical isolate	AMP, AMP/SUL, FOX, GEN, LEV, MOX, and OXA	This study
*S. aureus* H2	Clinical isolate	Resistant to COX, CFT, DAL, DPT, ERY, LEV, MOX, OXA, and TDZ	This study
*S. epidermidis* ATCC 35984	Reference strain	Resistant to CLI, ERY, GEN, OXA	Mazzantini et al., 2024a
*S. epidermidis* CI-1	Clinical isolate	Susceptible to all the tested antibiotics	Mazzantini et al., 2024a
*S. epidermidis* CI-2	Clinical isolate	Resistant to CLI, ERY, FUS, GEN, LEV, MOX, OXA, TPL, T/S, and VAN	Mazzantini et al., 2024a
*S. epidermidis* ME120121	Clinical isolate	Resistant to AMP, CIP, FOS, FUS, GEN, LEV, MOX, OXA, TOB, and T/S	This study

a AMP, ampicillin; AMP/SUL, ampicillin/sulbactam; CIP, ciprofloxacin; CLI, clindamycin; COX, cefoxitin; CFT, ceftobiprole; DAL, dalbavancin; DPT, daptomycin; ERY, erythromycin; FOF, fosfomycin; FOX, cefoxitin; FUS, fusidic acid; GEN, gentamicin; LEV, levofloxacin; MOX, moxifloxacin; OXA, oxacillin; PEN, penicillin; TDZ, tedizolid; TET, tetracycline; TOB, tobramycin; T/S, trimethoprim/sulfamethoxazole; VAN, vancomycin.

Depending on the experiments, strains were cultured in Mueller-Hinton broth (MHB, Thermo Fisher Scientific, Waltham, USA), Tryptone Soy Broth (TSB, ITW Reagents, Milan, Italy), or TSB supplemented with 0.25% (w/v) glucose (TSBG). When required, media were solidified by adding 1.5% (w/v) bacteriological agar.

### 2.2 Chemicals

**Figure 1:**
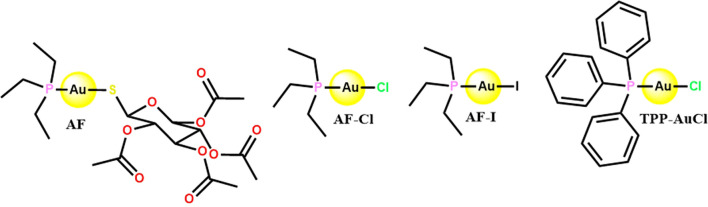
Chemical structures of the gold(I) complexes AF, AF-Cl, AF-I, and TPP-AuCl. This figure was created using ChemDraw (PerkinElmer, Waltham, USA).

The structures of the gold-compounds (AF, AF-Cl, AF-I, and TPP-AuCl) used in this study are shown in **Figure 1**. AF and AF-Cl were purchased from Sigma-Aldrich (Missouri, USA). AF-I and TPP-AuCl were synthesized according to methodologies reported in the literature (Baenziger et al., 1976; Marzo et al., 2018). The compounds were dissolved in dimethyl sulfoxide (DMSO, ITW Reagents, Milan, Italy) to a final concentration of 10 mg/mL and stored at – 20 °C until use.

### 2.3 Evaluation of the minimal inhibitory concentration

The gold-compounds were two-fold serially diluted in MHB in 96-well polystyrene microplates (Carlo Erba, Milan, Italy). Colonies were dissolved in 0.85% NaCl solution to 0.5 McFarland (~1.5×10^8^ CFU/mL) and the suspension was diluted in fresh MHB to a cell concentration of ~1×10^6^ CFU/mL. Then, 100 µL of the suspension were inoculated in the prepared microplates to obtain a bacterial concentration of ~5×10^5^ CFU/mL. The final concentration of gold-compounds in the assays ranged from 8 to 0.004 µg/mL. Wells containing bacteria and sterile MHB were used as positive and negative controls, respectively. Microplates were incubated at 35 ± 1 °C for 18 ± 2 h and the Minimal Inhibitory Concentration (MIC), defined as the lowest antimicrobial dilution that inhibits bacterial growth, was visually determined (The European Committee on Antimicrobial Susceptibility Testing, 2024).

### 2.4 Cytotoxicity assay

Cytotoxicity of gold compounds was tested against human epithelial cell line A549 (LGC Standards, Milan, Italy) and spontaneously immortalized human keratinocyte line (HaCaT, Cytion GmbH, Eppelheim, Germany). Cells were cultured at 37 °C in a humidified atmosphere containing 5% CO_2_ in 96-wells culture plates containing Dulbecco’s Modified Eagle Medium (DMEM High Glucose, Euroclone, Milan, Italy) supplemented with 10% fetal bovine serum and 2 mM L-glutamine (complete DMEM) to reach 90-100% confluence. Following washing to remove non-adherent cells, 150 µL of AF, AF-Cl, AF-I, and TPP-AuCl were added. The concentrations of the compounds in the assay ranged from 64 to 1-2 µg/mL. DMEM and Triton X 0.1% were added as control for cell viability and cytotoxicity, respectively. Then, plates were incubated for 24h at 37 °C in a humidified atmosphere containing 5% CO_2_. After supernatant has been removed, cells were subjected to the WST-1 assay (Roche, Basel, Switzerland) according to manufacturer’s instruction. The optical density at 620 nm (OD_620_) and at 450 nm (OD_450_) was measured and the percentage (%) of viability calculated. The concentration of each compound that kills 50% of cells (IC_50_) was determined using the Quest Graph™ IC_50_ calculator (AAT Bioquest, Inc https://www.aatbio.com/tools/ic50-calculator).

### 2.5 Time-kill assays

*S. aureus* W4 and *S. epidermidis* CI-2 were used for testing the time-kill kinetics of AF-Cl. Bacteria (~10^5^ CFU/mL) were suspended in MHB as control (i.e., CTRL) and in MHB containing AF-Cl at the MIC (0.063 µg/mL) and 2×MIC (0.125 µg/mL). Cultures were incubated at 37 °C for up to 24 h and subjected to plate count after 1, 3, 6, and 24 h of incubation. Plates were incubated at 37 °C for 24 h and the Log_10_ CFU/mL determined.

### 2.6 Inhibition of biofilm formation

*S. aureus* W4 and *S. aureus* H2 strains were grown overnight at 37 °C in 5 mL of TSB and TSBG, respectively. Cells were diluted 1:100 in fresh medium containing sub-inhibitory AF-Cl concentrations ranging from 0.25 to 0.0313 µg/mL. Cells without AF-Cl were used as CTRL. Two hundred µL of each suspension were dispensed in flat-bottom polystyrene 96-well microplates (Carlo Erba, Milan, Italy). AF-Cl at concentrations ranging from 0.5 to 0.0313 µg/mL without bacteria was included as negative controls in the assay. Microplates were incubated statically at 37 °C for 24 h. After that the medium containing non-adherent bacteria was removed, wells were washed three times with phosphate buffered saline (PBS; 1 M KH_2_PO_4_, 1 M K_2_HPO_4_, 5 M NaCl, pH 7.2). The biofilm biomass was quantified by the Crystal Violet (CV) assay as previously described (Mazzantini et al., 2024a). The percentage (%) of biofilm inhibition was calculated as follows (Liu et al., 2023):


$$Biofilm\;inhibition\;\left(\%\right)=100\times \frac{OD_{570\;CTRL}-OD_{570\;Treatment}}{OD_{570\;CTRL}}$$

### 2.7 Eradication of pre-formed (24h-old) biofilms

*S. aureus* W4 and *S. aureus* H2 strains were grown overnight at 37 °C in 5 mL of TSB and TSBG, respectively. After being diluted 1:100 in fresh medium, 200 µL of each suspension were dispensed into flat-bottom polystyrene 96-well polystyrene microplates (Carlo Erba, Milan, Italy). Wells containing media without bacteria were used as negative controls. Microplates were incubated at 37 °C for 24 h. Supernatants were removed and 200 µL of fresh medium (i.e., CTRL) or 200 µL of AF-Cl at concentrations ranging from 16 to 0.5 µg/mL were added. Microplates were incubated statically for additional 24 h and the CV assay was used to quantify the biofilm biomass (Mazzantini et al., 2024a). The percentage (%) of biofilm eradication was calculated as follows:


$$Biofilm\;eradication\;\left(\%\right)=100\times \frac{OD_{570\;CTRL}-OD_{570\;Treatment}}{OD_{570\;CTRL}}$$

To quantify the number of biofilm-associated bacteria, biofilms were mechanically detached from wells using sterile tips and transferred to 1 mL of PBS (Mazzantini et al., 2024a). Suspensions were vigorously vortexed for 30 seconds (s), sonicated for 30 s using a water bath sonicator (Ultrasonic cleaner, VWR), and vortexed for an additional 30 s. After being diluted in PBS, aliquots were seeded on agar plates for CFU counts. The Minimum Biofilm Bactericidal Concentration (MBBC), defined as the lowest concentration of AF-Cl required to kill the 99.99% (i.e., 3 log CFU/mL) of biofilm-embedded cells was determined (Macià et al., 2014).

### 2.8 Scanning electron microscopy of biofilms

Overnight cultures of *S. aureus* H2 were diluted 1:100 in 5 mL of TSBG and 600 µL dispensed into the wells of flat-bottom polystyrene 24-well microplates (Sarstedt, Nümbrecht, Germany), containing sterile glass slides with 11 mm of diameter. Microplates were incubated at 37 °C for 24 h. Supernatants were removed, and biofilms were treated with 600 µL of AF-Cl at concentrations of 4 and 8 µg/mL. Untreated biofilms served as CTRL. After 24h of incubation at 37 °C, glass slides were washed three times with PBS and biofilms fixed with 2.5% glutaraldehyde (Merck, Darmstadt, Germany) at 4 °C for 2.5h. Samples were dehydrated with absolute ethanol for 30 min, dried under a flow laminar hood overnight, and examined by field emission SEM FEI Quanta 450 FEG from the Center for Instrument Sharing of the University of Pisa, Italy (CISUP). Images were acquired from randomly selected areas on each sample at different magnifications.

### 2.9 Preparation of culture supernatants

*S. aureus* H2 was inoculated in 5 mL of TSB and grown overnight at 37 °C. The next day, 300 µL of the cultures were inoculated in 60 ml of fresh TSB (i.e., untreated control) and TSB containing 0.125 µg/mL of AF-Cl and were grown for 24h a 37 °C. Culture supernatants were collected by centrifuging cultures at 3,870 × g and were filtered using 0.22 μm filters to completely remove bacterial cells. Samples were maintained at -20 °C until use.

### 2.10 Hemolysis assay

Hemolysis assay was performed as previously described (Mazzantini et al., 2024b). Red blood cells (RBCs) were collected from healthy volunteers in accordance with the Declaration of Helsinki. The protocol was approved by the local Ethical Committee (Comitato Etico Area Vasta Nord-Ovest, CEAVNO, Protocol 34743, 28 June 2018). Briefly, 100 µL of bacterial supernatants were mixed with 100 µL of 1% RBCs into 96-well round bottom microplates (Carlo Erba, Milan, Italy) and incubated for 1h at 37 °C. RBCs mixed with PBS and RBCs mixed with 10% Triton X-100 (Merck KGaA, Darmstadt, Germany) were used as negative (0% lysis) and positive (100% lysis) controls in the assay, respectively. RBCs were precipitated by centrifuging at 1,700 × g for 5 min at 4 °C and 100 µL of supernatants were transferred to new microplates (Carlo Erba, Milan, Italy). The optical density at 405 nm (OD_405_) was measured using the Multiskan™ FC Microplate Photometer (Thermo Fisher Scientific, Waltham, USA) and the percentage of hemolysis was calculated according to the formula:


$$Hemolysis\;\left(\%\right)=100\times \frac{OD_{405\;Supernatant}-OD_{405\;Negative\;control}}{OD_{405\;Positive\;control}-OD_{405\;negative\;control}}$$

### 2.11 Quantitative determination of phospholipases activity

Culture supernatants were two-fold serially diluted in Tris Buffered Saline (8 g/L NaCl, 0.2 g/L KCl, 3g/L Tris Base, pH 7.5) and 10 µL were subjected to a gel-diffusion assay as previously described (Ghelardi et al., 2002). Different dilutions of phospholipase C (Merck KGaA, Darmstadt, Germany) were used to generate a standard calibration curve for the quantification of phospholipases activity. Glass slides were incubated for 24h at 37 °C in a humidified chamber and the diameter of precipitation halos was measured.

### 2.12 Statistical analysis

Experiments were repeated three times in separate days. Quantitative data were expressed as the mean ± standard error of the mean (SEM). All statistical analyses were performed using GraphPad Prism (version 8.0.2, Dotmatics, Boston, MA, USA). Depending on the experiment, the Student’s t-test for unpaired data or the One-Way ANOVA for independent data with Dunnett’s multiple comparisons test was applied, by setting the CTRL values as control group. A two-tailed p-value (p)< 0.05 was considered significant.

## 3 Results

### 3.1 Anti-staphylococcal activity and cytotoxicity of AF, AF-Cl, AF-I, and TPP-AuCl

The aim of the study was to select the most promising gold compound in the series for further investigation. To this end, we screened AF and its three analogues (i.e. AF-Cl, AF-I, and TPP-AuCl; **Figure 1**) for their anti-staphylococcal activity and cytotoxicity toward human cells.

The antimicrobial activity of the gold(I) compounds was tested against a panel of *S. aureus* and *S. epidermidis* strains, including reference and clinical strains with different antibiotic resistance profiles (**Table 1**). As shown in **Table 2**, all the compounds exhibited antimicrobial activity against both antibiotic-susceptible and -resistant staphylococci, with minimum inhibitory concentrations (MIC) ranging from 0.063 to 4 µg/mL. Among the tested compounds, AF-Cl and AF-I were the most active, with MIC values of 0.063–0.125 µg/mL against all staphylococci. In contrast, TPP-AuCl displayed the lowest activity, with MIC values of 0.5–4 µg/mL.

**Table 2:** Anti-staphylococcal activity of AF, AF-Cl, AF-I, and TPP-AuCl tested by broth microdilution.

	MIC^a^			
Bacterial strain	AF	AF-Cl	AF-I	TPP-AuCl
*S. aureus* ATCC 6538	0.25 (0.36)	0.125 (0.35)	0.125 (0.28)	1 (2.02)
*S. aureus* W4	0.25 (0.36)	0.063 (0.18)	0.125 (0.28)	1 (2.02)
*S. aureus* H1	0.125 (0.18)	0.031 (0.08)	0.063 (0.14)	4 (8.08)
*S. aureus* H2	0.125 (0.18)	0.063 (0.18)	0.125 (0.28)	0.5 (1.01)
*S. epidermidis* ATCC 35984	0.25 (0.36)	0.063 (0.18)	0.125 (0.28)	1 (2.02)
*S. epidermidis* CI-1	0.125 (0.18)	0.031-0.063 (0.08-0.18)	0.063 (0.14)	1 (2.02)
*S. epidermidis* CI-2	0.125 (0.18)	0.063 (0.18)	0.063 (0.14)	1 (2.02)
*S. epidermidis* ME120121	0.125 (0.18)	0.063 (0.18)	0.063 (0.14)	4 (8.08)

a MIC values are expressed as µg/mL (µM).

**Figure 2:**
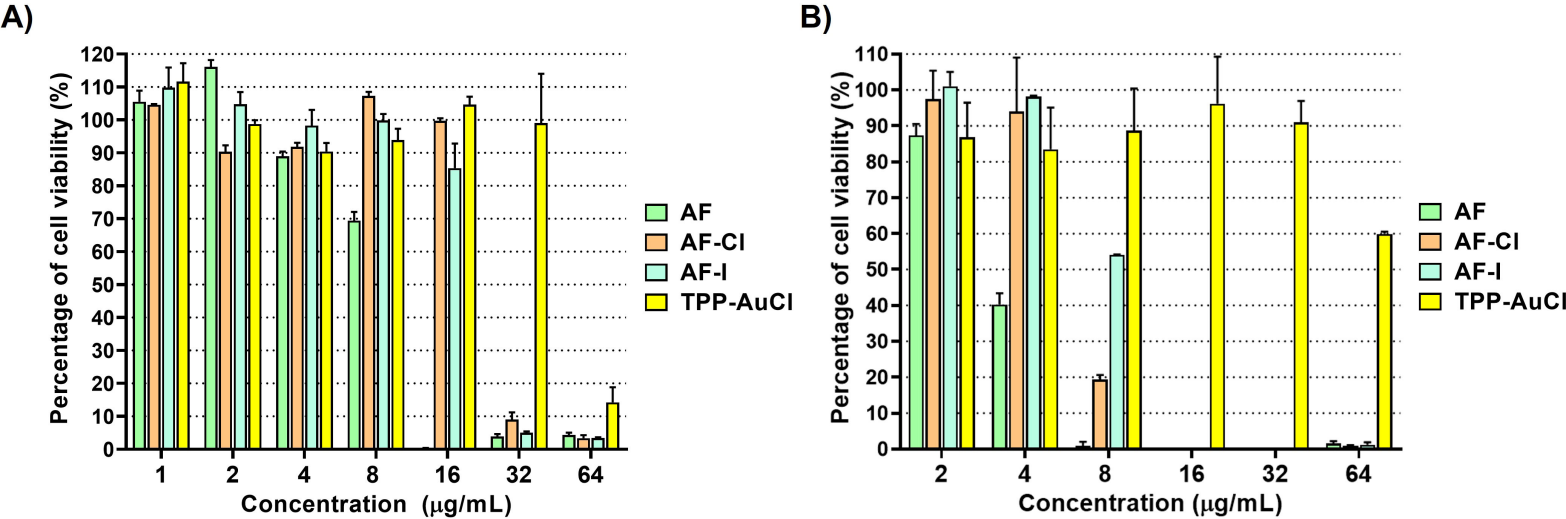
Cytotoxicity of gold-compounds on A549 human epithelial cell line **(A)** and the spontaneously immortalized human keratinocyte line HaCaT **(B)**. Cytotoxicity was expressed as percentage of cell viability (%). AF, green bars; AF-Cl, orange bars; AF-I, blue bars; TPP-AuCl, yellow bars.

The cytotoxic effects of AF, AF-Cl, AF-I and TPP-AuCl were evaluated against the human lung epithelial cell line A549 (**Figure 2A**) and the spontaneously immortalized human keratinocyte cell line HaCaT (**Figure 2B**). As shown in **Figures 2A**, TPP-AuCl showed the lowest cytotoxicity on A549 cells, since it did not impact cell viability at concentrations as high as 32 µg/mL (IC_50_: 54.24 ± 2.61 µg/mL). AF-Cl did not significantly affect the viability of A549 cells until 16 µg/mL, while displayed marked cytotoxic effects at higher concentrations (IC_50_: 21.60 ± 0.96 µg/mL). AF-I was not cytotoxic on A549 cells until 8 µg/mL but displayed progressive cytotoxicity starting from 16 µg/mL (IC_50_: 18.08 ± 0.16 µg/mL). Of the tested compounds, AF displayed the highest cytotoxicity. Indeed, the drug caused a reduction in A549 viability of ~11% and ~30% at concentrations of 4 and 8 µg/mL, respectively, and killed almost all cells starting from 16 µg/mL (**Figure 2A**; IC_50_: 8.84 ± 0.12 µg/mL).

A similar trend in cytotoxicity was obtained toward HaCaT (**Figure 2A**), although this cell line resulted more susceptible to AF, AF-Cl, and AF-I than A549 cells. In fact, AF-Cl and AF-I showed marked cytotoxic effects starting from 8 µg/mL, while AF induced a ~60% reduction in cell viability starting from 4 µg/mL. The calculated IC_50_ for AF, AF-Cl, AF-I, and TPP-AuCl were 3.98 ± 0.11, 7.17 ± 0.29, 8.10 ± 0.04, and 71.30 ± 0.17 µg/mL, respectively.

Overall, our findings highlighted AF-Cl as the most promising candidate due to its high antimicrobial activity and low cytotoxicity. Therefore, the anti-staphylococcal activity of this compound was investigated further.

### 3.2 AF-Cl exerts a rapid bactericidal activity

**Figure 3:**
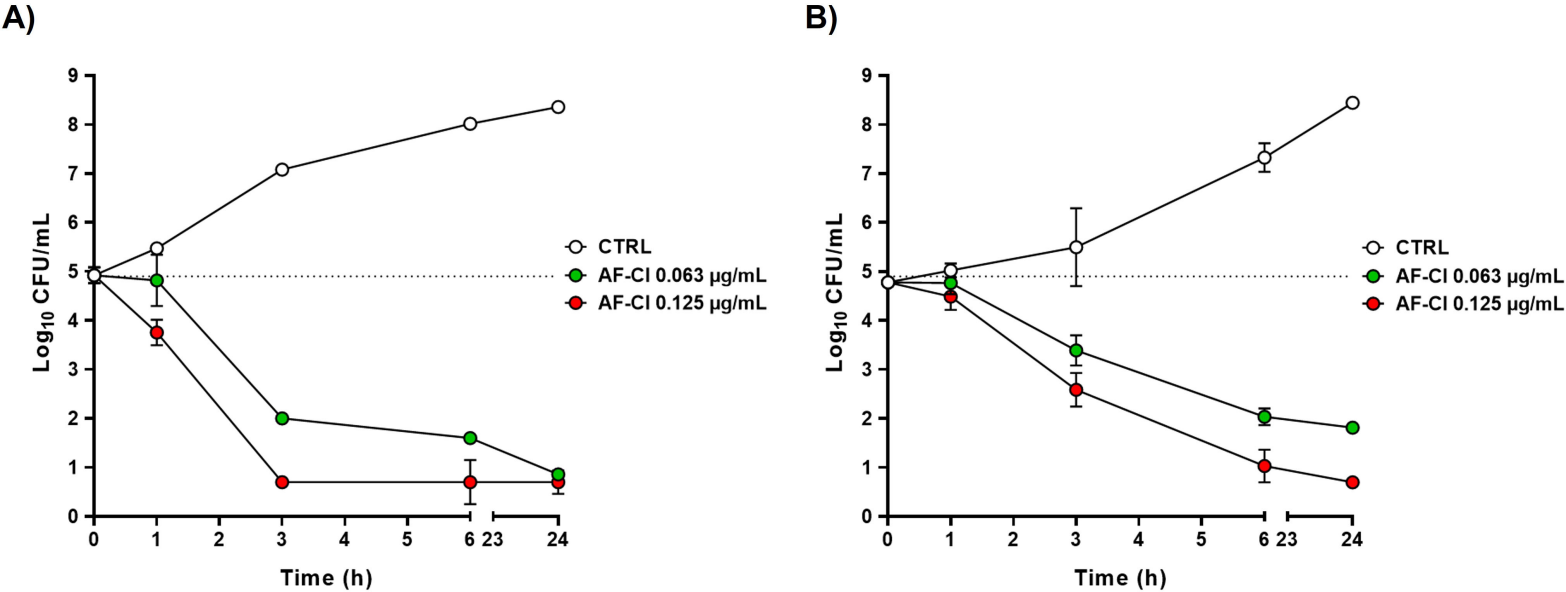
Killing kinetics of AF-Cl at the MIC (0.063 µg/mL) and 2×MIC (0.125 µg/mL) on *S. aureus* W4 **(A)** and *S. epidermidis* CI-2 **(B)**. White circles, untreated control (CTRL); green circles, AF-Cl at the MIC; red circles, AF-Cl at 2×MIC. The dotted line represents the initial Log_10_ CFU/mL.

To investigate the kill kinetics of AF-Cl, we performed time-kill experiments using two clinical isolates (*S. aureus* W4 and *S. epidermidis* CI-2) as model staphylococci. For these assays, the staphylococcal strains were treated with AF-Cl at an MIC of 0.063 µg/mL and a 2× MIC of 0.125 µg/mL in MHB for up to 24 hours, and the log CFU/mL was determined at different time points during the treatment. Bacteria in MHB were used CTRL. As shown in **Figure 3**, a progressive reduction in Log CFU/mL over time was evident for both strains treated with AF-Cl. At a concentration of 0.063 µg/mL, the compound induced a ~3 Log decrease in the number of viable *S. aureus* cells after 3 hours of incubation (**Figure 3A**). When AF-Cl was used at a concentration of 0.125 µg/mL, a reduction of more than 4- Log in the number of CFU was observed at the same time point. Regarding *S. epidermidis* CI-2 (**Figure 3B**), a 4-Log reduction in the number of CFU/mL was observed after 6 hours of incubation using 0.125 µg/mL of AF-Cl. Overall, these findings suggest that the compound exhibits rapid bactericidal activity.

### 3.3 Sub-inhibitory concentrations of AF-Cl reduce *S. aureus* biofilm formation

**Figure 4:**
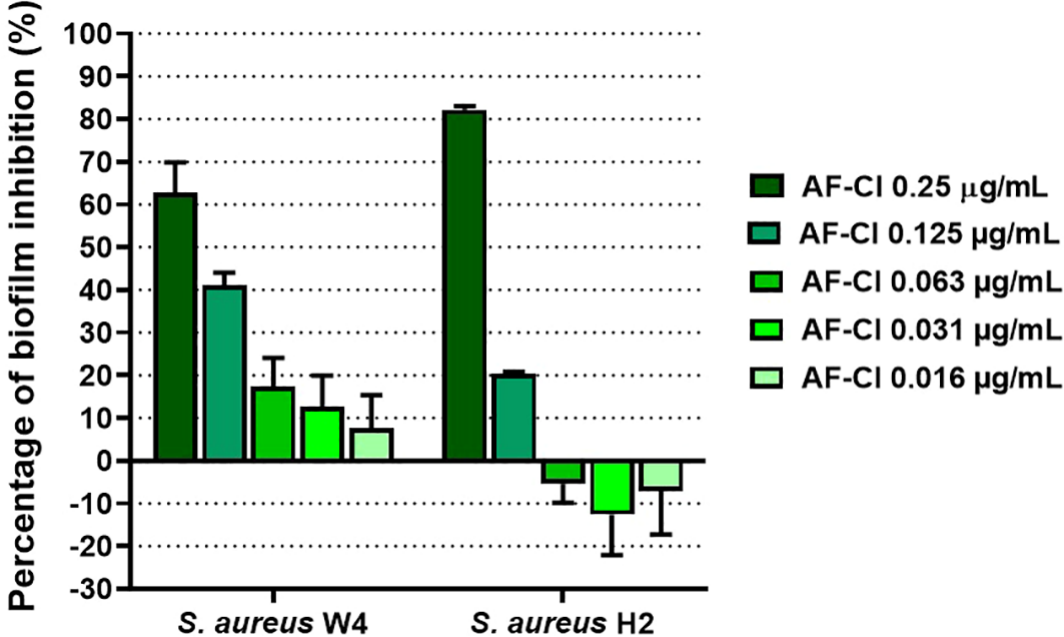
Effect of sub-inhibitory AF-Cl concentrations on *S. aureus* W4 and *S. aureus* H2 biofilm formation expressed as percentage of biofilm inhibition (%). The intensity of the green bars directly correlates with AF-Cl concentrations: (i.e., dark green, highest AF-Cl concentration; light green, lowest AF-Cl concentration).

Considering the critical role that biofilms play in staphylococcal infections (Beenken and Smeltzer, 2025), we investigated whether AF-Cl could affect biofilm formation at sub-inhibitory concentrations. The anti-biofilm activity of the compound was tested on *S. aureus* H2 and *S. aureus* W4, both of which were capable of forming a considerable amount of biofilms in TSB and TSBG, respectively. As the activity of antimicrobials can be affected by culture conditions (Bock et al., 2018), we first determined the MIC of AF-Cl towards *S. aureus* H2 and *S. aureus* W4 in TSB and TSBG, respectively. For both strains, the MIC of the compound increased to 0.5 µg/mL in these media. Both strains were then incubated in the presence of sub-inhibitory concentrations of AF-Cl ranging from 0.25 to 0.0313 µg/mL, after which their ability to produce biofilms was tested in comparison to CTRL using the CV assay. As regard to *S. aureus* W4, sub-inhibitory AF-Cl concentrations of 0.25 or 0.125 µg/mL significantly reduced the OD_570_ values obtained by the CV assay (p< 0.001 compared to CTRL, **Table 3**), with percentages of inhibition of 62.77 ± 7.10% and 41.03 ± 3.05%, respectively (**Figure 4**).

**Table 3:** Effect of sub-inhibitory AF-Cl concentrations on biofilm formation evaluated by the CV assay. Values (OD_570_) are expressed as the mean ± SEM.

AF-Cl concentration (µg/mL)	*S. aureus* W4	*S. aureus* H2
0.25	0.283 ± 0.028^a^	0.139 ± 0.016^b^
0.125	0.460 ± 0.018^a^	0.618 ± 0.042^c^
0.063	0.641 ± 0.008	0.822 ± 0.087
0.032	0.681 ± 0.047	0.875 ± 0.106
0.016	0.718 ± 0.038	0.841 ± 0.130
0 (CTRL)	0.787 ± 0.069	0.755 ± 0.052

a p< 0.001.

b p< 0.01.

c p< 0.05 compared to the corresponding CTRL.

These concentrations also significantly reduced biofilm production by *S. aureus* H2 (p< 0.01 and p< 0.05 compared to CTRL; **Table 3**), with percentages of inhibition of 82.18 ± 0.85% and 20.28 ± 0.53%, respectively (**Figure 4**). Overall, these results highlight the ability of AF-Cl to reduce *S. aureus* biofilm formation at sub-inhibitory concentrations.

### 3.4 AF-Cl eradicates mature (24h-old) staphylococcal biofilms

**Figure 5:**
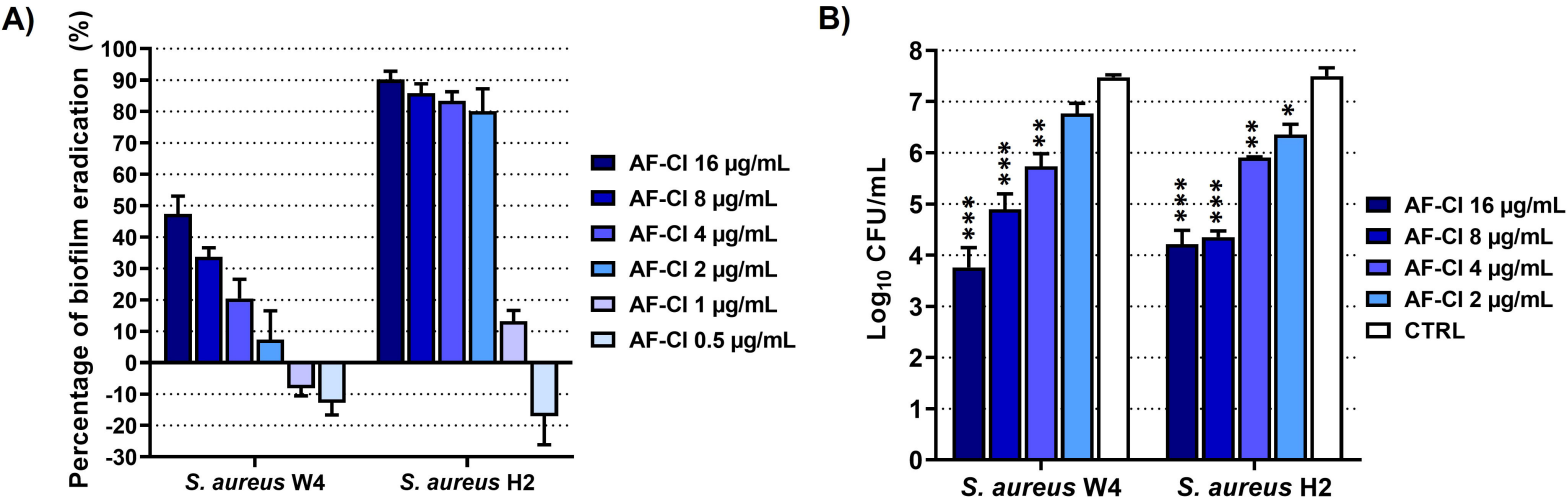
Eradicating effect of AF-Cl at concentrations equal and higher than the MIC on mature biofilms formed by *S. aureus* W4 and *S. aureus* H2. **(A)** Percentage of biofilm eradication (%). The intensity of blue bars directly correlates with AF-Cl concentration: (i.e., dark blue: the highest AF-Cl concentration, light blue: the lowest AF-Cl concentration). **(B)** Quantification of biofilm-associated bacteria by plate count. White bars: untreated controls (CTRL); the intensity of blue bars directly correlates with AF-Cl concentration: (i.e., dark blue: the highest AF-Cl concentration, light blue: the lowest AF-Cl concentration). The One-Way ANOVA for independent data with Dunnett’s multiple comparisons test was applied, by setting the CTRL as control group. *p< 0.05, **p< 0.01, and ***p< 0.001 compared to CTRL.

To test the eradicating activity of AF-Cl on mature biofilms, 24h-old biofilms of *S. aureus* W4 and *S. aureus* H2 were treated with concentrations of the compound ranging from 0.5 (i.e., the MIC in TSB and TSBG) to 16 µg/mL, which represented the highest AF-Cl concentration that did not displayed cytotoxic effects on A549 cells. As shown in **Figures 5A**, AF-Cl significantly reduced the biomass of biofilms formed by both strains compared to the respective CTRLs. The most prominent eradicating effect was observed against the biofilms formed by *S. aureus* H2. In fact, the compound significantly reduced the OD_570_ values obtained by the CV assay starting from 2 µg/mL of concentration (p< 0.001 compared to CTRL, **Table 4**), with percentages of eradication of 80.07 ± 7.13%, 83.38 ± 2.89%, 85.79 ± 3.03%, and 90.20 ± 2.62% when used at concentrations of 2, 4, 8, and 16 µg/mL, respectively (**Figure 5A**).

**Table 4:** Effect of AF-Cl concentrations equal and higher than the MIC on mature biofilm evaluated by the CV assay. Values (OD_570_) are expressed as the mean ± SEM.

AF-Cl concentration (µg/mL)	*S. aureus* W4	*S. aureus* H2
16	0.631 ± 0.055^a^	0.141 ± 0.034^c^
8	0.800 ± 0.052^a^	0.208 ± 0.046^c^
4	0.954 ± 0.048^c^	0.244 ± 0.045^c^
2	1.113 ± 0.096	0.293 ± 0.109^c^
1	1.302 ± 0.025	1.205 ± 0.048
0.5	1.357 ± 0.022	1.624 ± 0.127
0 (CTRL)	1.206 ± 0.039	1.492 ± 0.090

a p< 0.001 and.

c p< 0.05 compared to the corresponding CTRL.

Starting from 4 µg/mL (**Table 4**), AF-Cl significantly reduced the biomass of biofilms formed by *S. aureus* W4, with percentages of eradication of 20.49 ± 6.11%, 33.72 ± 2.86%, and 47.39 ± 5.69% at concentrations of 4, 8, and 16 µg/mL, respectively (**Figure 5A**).

To evaluate the impact of AF-Cl on the viability of biofilm-associated cells, we quantified the number of living bacteria contained in treated biofilms by plate count. As shown in **Figures 5B**, all the concentrations of the compound that were able to reduce the biofilm biomass in the CV assay (i.e., 2, 4, 8, and 16 µg/mL for *S. aureus* H2 and 4, 8, and 16 µg/mL for *S. aureus* W4) significantly affected the viability of both strains in a dose-dependent way. A reduction of at least 3-Log in the number of CFU/mL (i.e., MBBC) was observed when biofilms of *S. aureus* W4 were treated with 16 µg/mL of AF-Cl. Notably, AF-Cl exerted its bactericidal effect on biofilms formed by *S. aureus* H2 starting from 8 µg/mL (i.e., MBBC). Overall, these findings highlight the ability of the compound to eradicate mature *S. aureus* biofilms by killing biofilm-embedded cells.

### 3.5 AF-Cl induced cell dispersion and massive morphological alterations

**Figure 6:**
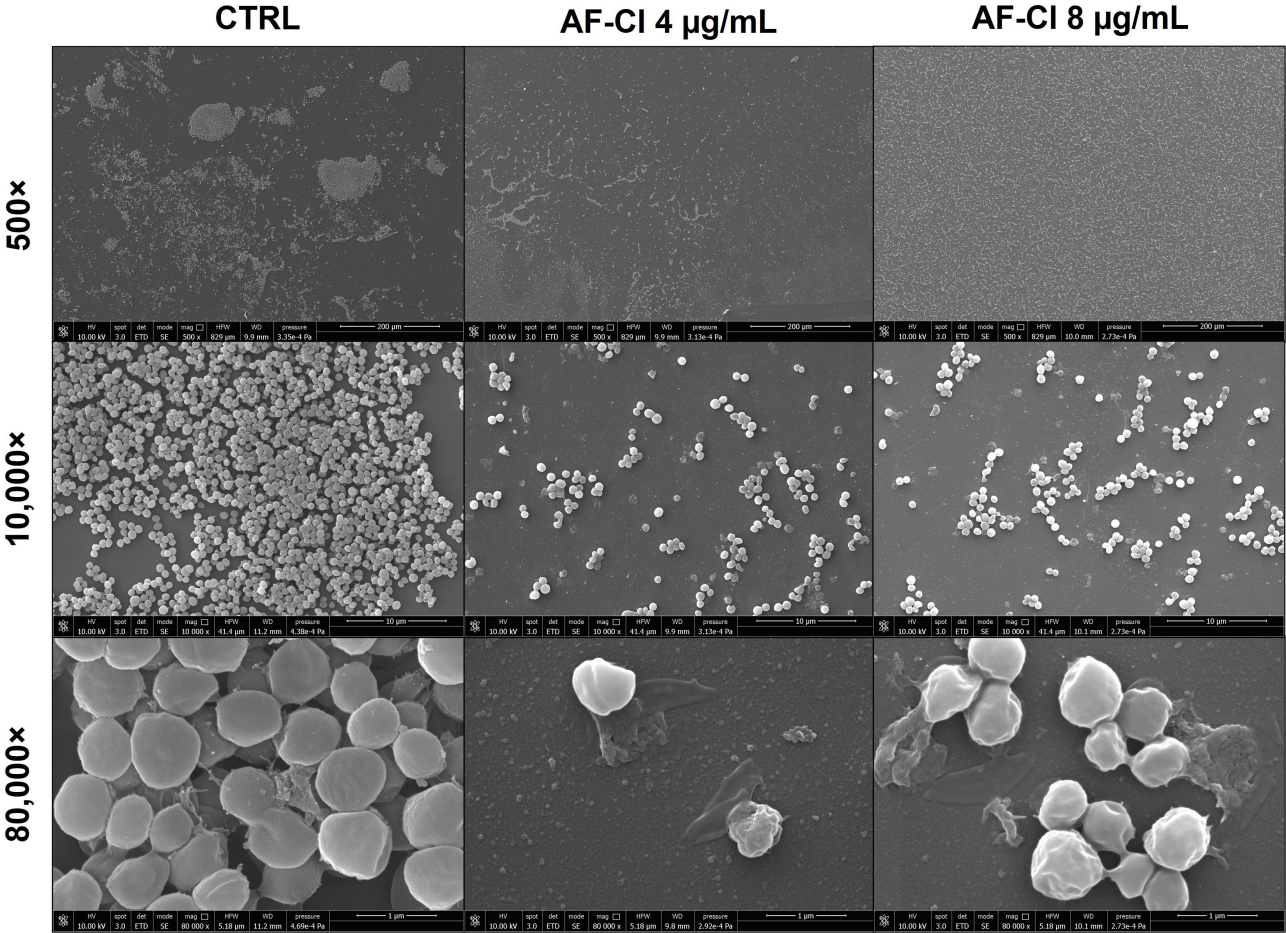
SEM images of untreated (CTRL) and treated biofilms (4 and 8 µg/mL) of *S. aureus* H2 at different magnifications (i.e., 500×, 20,000×, and 80,000×).

The effect of AF-Cl at MBBC (8 µg/mL) and sub-MBBC (4 µg/mL) concentrations on *S. aureus* H2 biofilms, found to be more susceptible than those formed by *S. aureus* W4, were analyzed by SEM. As shown in **Figure 6**, biofilms treated with AF-Cl at both concentrations exhibited significant ultrastructural differences compared to CTRL. Untreated biofilms consisted of regularly shaped, spherical cells tightly adhered to each other, forming well-organized, multilayered structures. In contrast, AF-Cl treatment at 4 and 8 µg/mL induced a dose-dependent increase in cell dispersion. At 80000× magnification, treated biofilms displayed significant morphological alterations, including cell damage and release of intracellular material, particularly at 8 µg/mL, suggesting bacterial killing. Taken together, these results support the ability of AF-Cl to eradicate mature staphylococcal biofilms and to kill biofilm-associated cells.

### 3.6 Anti-virulence effect of AF-Cl

**Figure 7:**
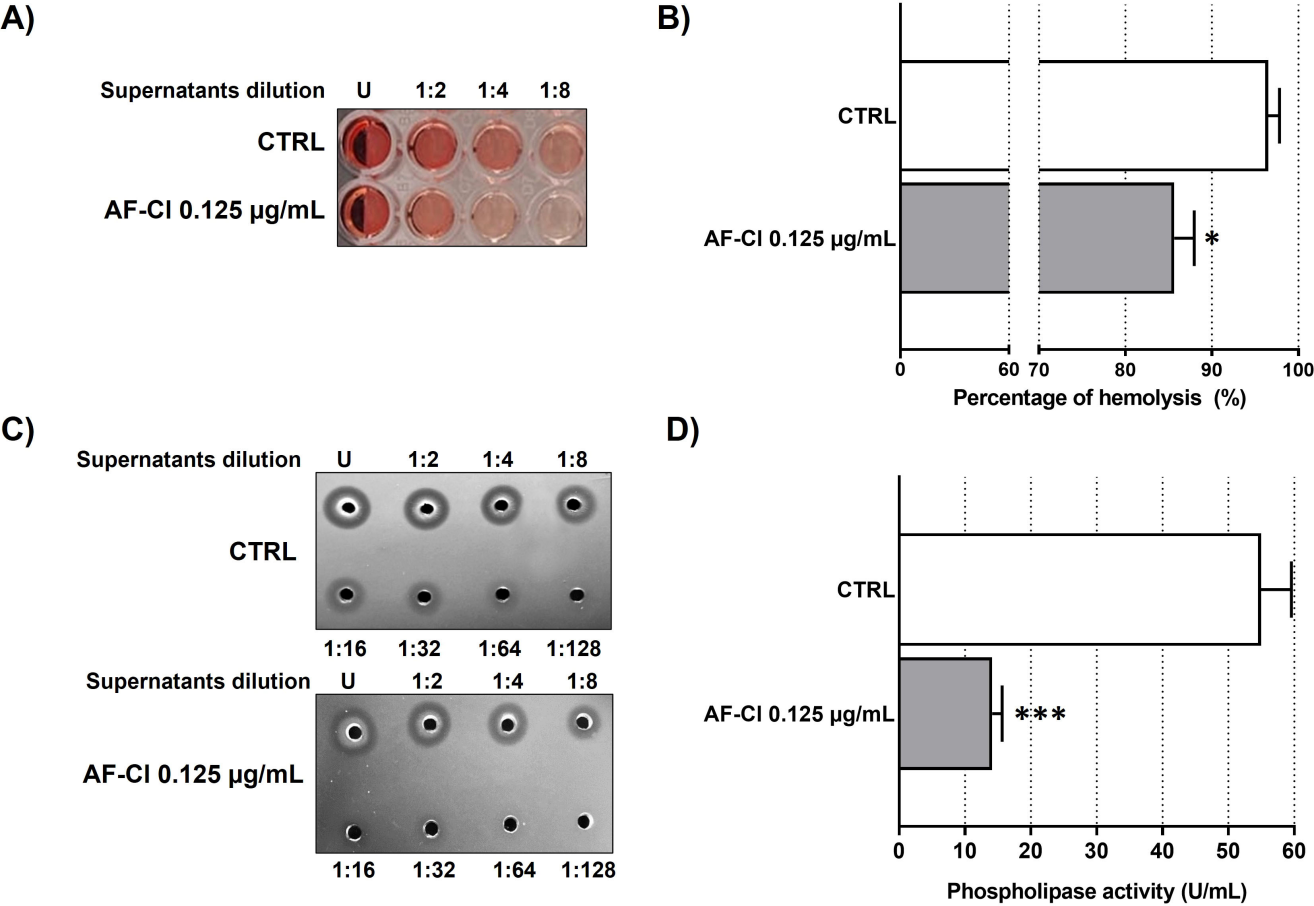
Anti-virulence effect of AF-Cl at 0.125 µg/mL on *S. aureus* H2. **(A)** Hemolytic activity of culture supernatants obtained from untreated (CTRL) and AF-Cl treated cells. Undiluted (U) and 2-, 4-, and 8-fold diluted supernatants were used. **(B)** Percentage of hemolysis (%) of culture supernatants obtained from untreated (CTRL, white bars) and AF-Cl treated (grey bars) cells. **(C)** Agar diffusion assay of culture supernatants obtained from untreated (CTRL) and AF-Cl treated cells. Undiluted (U) and diluted supernatants (from 1:2 to 1:128) were used. **(D)** Phospholipase activity (U/mL) of culture supernatants obtained from untreated (CTRL, white bars) and AF-Cl treated (grey bars) cells. Quantitative data were analyzed using the Student’s t-test for unpaired data. *p< 0.05 and ***p< 0.001 compared to CTRL.

The anti-virulence effect of AF-Cl was investigated on *S. aureus* H2 as this strain resulted the highest producer of hemolysins and phospholipases among the tested strains. *S. aureus* H2 cells were treated with AF-Cl 0.125 µg/mL for 24h and the amount of hemolysins and phospholipases in culture supernatants was quantified through hemolysis and agar diffusion assays, respectively, in comparison to CTRL. At this time point, no differences in the number of CFU/mL between treated (1.59 ± 0.03×10^9^ CFU/mL) and untreated cells (2.50 ± 0.62×10^9^ CFU/mL) were observed (p > 0.05). Supernatants obtained from AF-Cl-treated cells displayed a lower hemolytic activity than CTRL (**Figure 7A**) and a ~11% reduction in the percentage of hemolysis was registered (**Figure 7B**).

In addition, as shown in **Figures 7C, D**, AF-Cl was found able to reduce the amount of secreted phospholipases. Overall, these findings indicate that the compound exerts anti-virulence effects when used at sub-inhibitory concentrations.

## 4 Discussion

The positive effects of gold on human health have been recognized for 5,000 years. Its use in various fields of medicine, including dentistry, traditional Chinese medicine, dermatology, and rheumatology, is well established (Balfourier et al., 2020). In recent decades, gold(I)-based complexes, including AF and its analogues, have attracted considerable interest as promising agents for treating microbial infections due to their broad-spectrum antimicrobial, anti-biofilm, and anti-virulence activities (AbdelKhalek et al., 2018, 2019; Abutaleb and Seleem, 2020; Coscione et al., 2024; Ferretti et al., 2025; Ma et al., 2025; Marques et al., 2023; Marzo et al., 2018; Yam et al., 2023). In this study, we screened a series of AF analogues to identify the compound with the strongest antimicrobial activity. To evaluate their efficacy in treating infections caused by antibiotic-resistant staphylococci, antimicrobial assays were conducted against a panel of reference and clinical strains exhibiting diverse antibiotic resistance profiles. AF-Cl and AF-I exhibited stronger anti-staphylococcal activity than AF, with comparable MIC values across all tested strains. These results are consistent with those of a previous study (Marzo et al., 2018), which investigated the antimicrobial activity of AF-Cl and AF-I against *S. aureus* ATCC 25923 and *S. epidermidis* FI-1. The greater antimicrobial activity of AF-Cl and AF-I compared to AF may be due to their ability to release chloride and iodide ions, producing the [AuPEt_3_]^+^ fragment. This positively charged fragment can interact more easily with the bacterial surface through electrostatic interactions, promoting internalization. In contrast, the synthesized TPP-AuCl exhibited lower antimicrobial activity than the other gold(I) complexes. We hypothesize that the presence of phenyl groups in TPP-AuCl could hinder interaction with the cell surface, possibly reducing uptake and efficacy.

When selecting pharmaceutical candidates for human use, evaluating cytotoxicity is essential to exclude potential toxic effects on host cells. In this study, the cytotoxicity of gold compounds at concentrations above their MICs was primarily assessed using the human A549 lung carcinoma cell line, a widely accepted model for profiling drug-induced cytotoxicity and frequently employed in similar investigations (Barak et al., 2025; Kumar et al., 2025; Oyardi et al., 2022). In parallel, HaCaT keratinocytes were included to further examine the cytotoxic effects of the tested gold compounds.

Overall, HaCaT cells showed greater susceptibility to the compounds than A549 cells. This finding agrees with previous studies demonstrating that HaCaT cells are generally more sensitive to the cytotoxic action of various agents compared with A549 cells (Liu et al., 2012; Spagnoletti et al., 2016).

For all compounds, cytotoxic concentrations were substantially higher than the corresponding MICs. Moreover, all three AF analogues exhibited lower cytotoxicity than AF in both cell lines, with TPP-AuCl being the least cytotoxic. Specifically, AF caused approximately 30% and 100% cytotoxicity in A549 cells at 8 and 16 µg/mL, respectively, consistent with the results reported by Ferretti et al. (2025). Although AF is approved for clinical use at a dose of 6 mg/day (Capparelli et al., 2016), its cytotoxic effects on several tumor cell lines—including lung cancer cells—have been well documented, and it is currently undergoing evaluation as an anticancer agent (Giorgini et al., 2025; Marzo et al., 2017; Roder and Thomson, 2015). To expand the therapeutic applications of AF beyond oncology, structural analogues have been developed that exhibit markedly reduced cytotoxicity alongside improved antimicrobial activity (Chen et al., 2025; Epstein et al., 2019; Ferretti et al., 2025). Based on the antimicrobial and cytotoxicity profiles obtained in this study, AF-Cl was selected as the most promising candidate for further investigation, offering the best balance between anti-staphylococcal activity and cytotoxicity.

Investigation of the killing kinetics of AF-Cl, the most promising compound identified through antimicrobial and cytotoxicity screening, revealed bactericidal activity against both *S. aureus* and *S. epidermidis* within 6 hours. Notably, this activity was faster than that exhibited by AF against *Enterococcus faecium* (AbdelKhalek et al., 2018). Although the mechanism of action of AF-Cl is not completely elucidated yet, in light of recent literature (Tolbatov and Marrone, 2026) it is possible to hypothesize that it acts on one or more critical targets for bacterial survival.

The anti-biofilm and anti-virulence activity of the parent compound AF are well known (AbdelKhalek et al., 2018; Chen et al., 2023; Jang and Eom, 2020; Thangamani et al., 2016; She et al., 2019). Nevertheless, a recent computational and bioinformatic study indicated different interactors for AF and AF-Cl, thus suggesting a non-identical mechanism of action of these molecules (Tolbatov and Marrone, 2026). In fact, while AF has been shown to preferentially interact with the positively charged side chain of lysine, the indole scaffold of tryptophan was indicated as favorable interactor of AF-Cl (Tolbatov and Marrone, 2026).

*S. aureus* can form robust biofilms on abiotic surfaces, including prostheses, contact lenses, and orthopedic devices. The bacterium is responsible for a variety of challenging biofilm-associated infections in humans (Beenken and Smeltzer, 2025). These biofilms are much more resistant to antibiotics and host immune cells than planktonic bacteria, posing a significant health risk (Beenken and Smeltzer, 2025). Therefore, antimicrobials that can prevent biofilm formation and eradicate pre-established biofilms are highly desirable (Kalia et al., 2023). Previous studies have demonstrated the ability of AF and its derivatives (i.e., AF-Napx and AF-AcCys) to eradicate biofilms formed by *S. aureus* and *S. epidermidis* (Ferretti et al., 2025). Furthermore, AF has been found to exhibit anti-biofilm properties against itraconazole-resistant *Aspergillus fumigatus*, *Bacteroides fragilis*, *Enterococcus faecalis* and *E. faecium* (AbdelKhalek et al., 2018; Chen et al., 2023; Jang and Eom, 2020; She et al., 2019). In this study, we observed that AF-Cl reduces *S. aureus* biofilm formation at sub-inhibitory concentrations. This suggests that the compound may affect the attachment and/or aggregation of cells, thus reducing biofilm formation. The anti-biofilm capability of the compound could be advantageous *in vivo*, as it could counteract biofilm production and prevent biofilm-related infections. Additionally, we observed that concentrations of AF-Cl higher than the MIC can eradicate mature *S. aureus* biofilms by killing biofilm-embedded cells in a dose-dependent manner. Notably, the highest tested concentrations of AF-Cl (8 and 16 µg/mL) were bactericidal for biofilms, thus highlighting the potential of using the gold complex to eradicate mature biofilms *in vivo*.

Nowadays, scientific research focuses on developing therapeutic options with anti-virulence properties that can neutralize pathogens and minimize damage to the host, even at sub-inhibitory concentrations (Dickey et al., 2017). Thangamani et al. (2016) demonstrated that AF exhibits anti-virulence properties by reducing the production of certain *S. aureus* virulence factors, including α-hemolysins and Panton-Valentine leucocidin. We wondered whether AF-Cl could affect the secretion of hemolysins and phospholipases, which play a key role in *S. aureus* pathogenicity. Indeed, approximately 95% of *S. aureus* strains secrete α-hemolysin, which forms pores on the surfaces of erythrocytes, platelets, epithelial and endothelial cells, as well as B and T cells. This contributes to necrosis and systemic toxicity (Di Bella et al., 2025; Touaitia et al., 2025). Around 99% of *S. aureus* strains secrete γ-hemolysin, which exhibits hemolytic activity and is implicated in septic arthritis, pneumonia, endocarditis, and sepsis (Di Bella et al., 2025). *S. aureus* produces various phospholipases, including phosphatidylinositol-specific phospholipase C (PI-PLC) and β-hemolysin (also known as sphingomyelinase C), which hydrolyze phosphatidylinositol and sphingomyelin, respectively (Nakamura et al., 2020). PI-PLC plays an important role in *S. aureus* skin infections by enhancing the invasion of keratinocytes (Nakamura et al., 2020), while β-hemolysin modulates the host immune response, thereby exacerbating skin infections, abscesses and sepsis (Di Bella et al., 2025). It disrupts epithelial cells, erythrocytes, and leukocytes, triggering inflammation by activating the epidermal growth factor receptor (Jia et al., 2024). Furthermore, β-hemolysin has been shown to stimulate IFN-γ production by natural killer (NK) cells, suggesting a potential role in the development of chronic inflammatory diseases (Guan et al., 2021).

In this study, we found that exposing bacteria to a sub-inhibitory dose of AF-Cl significantly reduced the secretion of hemolysins and phospholipases. This highlights the anti-virulence activity of the compound, which could contribute to alleviating the symptoms of *S. aureus* diseases *in vivo*. Further research is needed to understand the role of AF-Cl in the synthesis and/or secretion of *S. aureus* virulence factors. Based on recent reports, we hypothesize that the compound may modulate gene expression, thereby affecting the activity of some transcriptional regulatory networks that control *S. aureus* virulence (Touaitia et al., 2025; Li et al., 2026). For instance, AF-Cl could directly inhibit the Accessory Gene Regulator (Agr) quorum sensing system, whose activity upregulates the production of hemolysins and phospholipases in this microbe (Touaitia et al., 2025). On the other hand, the compound could affect the Staphylococcal Accessory Regulator (Sar) system, in particular SarA, which control the transcription of some *S. aureus* virulence genes, including the same *agr* (Touaitia et al., 2025). Interestingly, a recent study highlighted the ability of AF to downregulate *sarA*, thus attenuating *S. aureus* virulence (Li et al., 2026).

## 5 Conclusion

In this study, we identified AF-Cl as a promising analogous of AF, exhibiting rapid and potent anti-staphylococcal activity, without any detectable cytotoxicity at concentrations exceeding its MIC In addition, to confirm the strong antimicrobial properties of AF-Cl, our findings demonstrate for the first time that this compound also exhibits significant anti-biofilm and anti-virulence activities. Indeed, when used at sub-inhibitory concentrations, AF-Cl appears to reduce biofilm formation and the secretion of hemolysins and phospholipases by *S. aureus*. Furthermore, non-cytotoxic concentrations of AF-Cl eradicate pre-formed *S. aureus* biofilms and kill embedded cells in a dose-dependent manner. Overall, our results highlight the potential of AF-Cl as valid anti-staphylococcal treatment. Further studies are needed to fully explore the antimicrobial mechanisms of AF-Cl and validate its suitability as anti-staphylococcal and anti-biofilm option also *in vivo*.

## Data Availability

The raw data supporting the conclusions of this article will be made available by the authors, without undue reservation.
